# Determinants of Digital Health Literacy Among Patients With Serious Mental Illness: Cross-Sectional Survey

**DOI:** 10.2196/88700

**Published:** 2026-04-15

**Authors:** Yi-Ju Chou, Kai-Jo Chiang, Hsin Huang, Hsin-An Chang, Yin-Ling Hung, Wen-Chii Tzeng

**Affiliations:** 1 Tri-Service General Hospital, Taipei City, Taiwan; 2 National Taipei University of Nursing and Health Science, Taipei City, Taiwan; 3 National Defense Medical University, No. 161, Sec 6, Min-quan E. Rd. Neihu Dist, Taipei City, 114201, Taiwan, 886 287923100 ext 18759

**Keywords:** attitude to computers, digital health, health literacy, internet use, mental disorders, self-efficacy

## Abstract

**Background:**

Individuals with serious mental illness increasingly use digital devices and the internet to access health information and services but often face challenges when navigating digital tools, which may limit the benefits they receive from online health resources and digital health care services.

**Objective:**

The objective of our study was to assess digital health literacy among individuals with serious mental illness and identify factors influencing this literacy.

**Methods:**

Participants were recruited, using convenience sampling, from 2 psychiatric clinics, 1 day-care center, and 4 halfway houses in Taipei, Taiwan, between May 2024 and February 2025. Self-reported data were collected using a survey that incorporated the eHealth Literacy Scale, the Attitudes Toward Computer/Internet Questionnaire, and the Mobile Device Proficiency Questionnaire. Generalized linear modeling was applied to identify factors associated with digital health literacy.

**Results:**

Among 255 participants included in the analysis, 83.5% (n=213) reported owning at least 1 digital device. Digital health literacy was significantly lower among individuals who reported greater perceived difficulty in using digital tools (*B*=−1.533, 95% CI −2.350 to −0.717; *P*<.001) and higher distrust in online information (*B*=−0.986, 95% CI −1.916 to −0.056; *P*=.04). By contrast, greater mobile device proficiency (*B*=0.144, 95% CI 0.008‐0.281; *P*=.04) and self-efficacy (*B*=1.777, 95% CI 0.376‐3.177; *P*=.01) were positively associated with digital health literacy.

**Conclusions:**

Despite widespread device ownership, digital health literacy was varied and generally suboptimal among patients with serious mental illness. Perceived difficulty and distrust emerged as major barriers; proficiency and self-efficacy facilitated higher literacy. These findings highlight the need for mental health professionals to integrate tailored digital skills training, confidence-building strategies, and ongoing support into digital health interventions for individuals with serious mental illnesses.

## Introduction

Rapid advancements in information and communication technology have provided an estimated 68.7% of the global population with internet access [1]. Consequently, digital health has become a core component of contemporary health care systems, enabling individuals to access, manage, and engage with health information and services [2]. The increasing incorporation of digital apps and platforms into health services has rendered navigation of these apps an essential competence to ensure equitable care access and support recovery-oriented practices for all patients, including individuals with serious mental illness.

Although most patients with serious mental illness own a digital device, substantial disparities persist in their device use proficiency and ability to access reliable online health information [3,4]. Digital technologies can support the alleviation of psychiatric symptoms and self-management [5]; however, their effectiveness largely depends on users’ digital health literacy or users’ capacities to seek, understand, evaluate, and apply information obtained from the internet or digital resources [6]. Inadequate literacy may result in digital exclusion, which entrenches current health inequities, limiting individuals’ ability to self-manage, participate in their own care [7], and make informed health-related decisions [8].

Digital health literacy can improve health outcomes, mitigate the digital divide, and reduce health inequalities [9]. However, patients with serious mental illnesses often demonstrate a lower capacity to use digital platforms compared with the general population [10]. These gaps may impede their ability to engage in person-centered, recovery-oriented care [7]. Health professionals maintain sustained therapeutic relationships with these patients and are therefore key to identifying and addressing digital health challenges. Yet, research on the specific determinants of digital health literacy among individuals with serious mental illnesses remains limited.

Digital health approaches have evolved from the earlier, narrower concept of eHealth, which focuses primarily on health care providers’ information and communication technology use. Digital health encompasses broader technological modalities, including mobile, remote, wearable, and artificial intelligence–based devices [9]. The eHealth Literacy 3.0 framework reconceptualizes literacy as the ability to effectively, safely, and meaningfully engage with digital technologies to achieve health goals [11]. This framework integrates information literacy (the ability to seek, find, understand, critically evaluate, and ethically create information), computer literacy (the capacity to adapt to emerging technologies), and media literacy, encompassing critical and reflective thinking that informs health-related decision-making. Accordingly, digital health literacy extends beyond basic operational skills to include higher-order competencies such as critical appraisal, data privacy awareness, and the interpretation of health information across diverse digital platforms [12].

Despite their conceptual distinction, the terms “digital health literacy” and “eHealth literacy” are often used interchangeably in the literature [9]. The eHealth Literacy Scale (eHEALS), developed in alignment with self-efficacy and social cognitive theory, is among the most widely used tools for the assessment of perceived ability to locate, evaluate, and apply online health information [6,13]. The Traditional Chinese version of this scale (eHEALS-TC) has demonstrated excellent psychometric properties in Taiwan, including high internal consistency and a stable unidimensional structure [14]. Its concise 8-item format minimizes respondent burden in clinical settings (including respondents with serious mental illness) and facilitates cross-national comparability [6,13].

Empirical studies have demonstrated substantial variability in eHEALS scores among individuals with serious mental illness. Moderate digital health literacy has been observed among individuals with schizophrenia in Finland (mean 27.05) and the United Kingdom (mean 26.0), whereas lower rates have been observed in Greece (mean 23.15) [15,16]. Internet users with bipolar disorder in the United States exhibited high scores (mean 31.7), exceeding those of both the general population and individuals with other mental or physical health conditions [17]. These findings suggest heterogeneity in digital health literacy across sociocultural contexts.

Sociodemographic and psychological determinants of digital health literacy include younger age, higher income, greater social support, and a higher education level [8,18,19]. Although younger age is typically associated with higher digital health literacy in the general population, evidence from individuals with bipolar disorder suggests that older age may predict higher eHEALS scores, possibly reflecting accumulated experience in their management of chronic conditions [17]. Psychological factors, such as high self-efficacy, positive attitudes toward technology, general health literacy, and trust in online information, are correlated with higher digital health literacy [20,21]. However, most studies of such factors have focused on segments of the general population, including internet users, university students, health care professionals, and older adults, limiting the applicability of these findings to individuals with serious mental illness.

Individuals with serious mental illness may encounter additional barriers affecting self-perceived digital health literacy. A qualitative study in Finland reported that these individuals experience difficulty accessing digital health services due to limited digital skills and capacity to navigate online platforms [22]. Similarly, a UK study highlighted forgetfulness, low motivation, and concerns regarding the replacement of face-to-face care as barriers to the use of digital mental health apps [23]. Limited awareness of available technologies has also been associated with low use of digital health resources among individuals with serious mental illness [4]. Furthermore, psychiatric symptoms may impede digital engagement. Concentration difficulties and depressive episodes can hinder the critical appraisal of online health information [16]. Additionally, cognitive impairments in attention, memory, and executive functioning may constrain information processing, credibility evaluation, and accurate self-assessment of digital competence [21].

Digital health literacy is increasingly recognized as a determinant of health equity [12]. Based on social cognitive theory and the eHealth Literacy 3.0 framework, digital health literacy can be conceptualized as a multidimensional construct shaped by structural resources, digital access and use patterns, and psychosocial mechanisms. It reflects the dynamic interaction between operational skills (eg, mobile device proficiency), attitudinal readiness (eg, interest in digital health), and confidence-related factors (eg, self-efficacy and trust in online information) [11,14]. Extending beyond abilities to locate and appraise information, digital health literacy encompasses safe, ethical, and goal-oriented engagement with digital technologies. This conceptual model informed the selection of study variables and the multivariable analytical approach.

Related research has primarily focused on the frequency of digital tool use, access to digital devices, or adoption of digital apps rather than on the determinants of digital health literacy itself. Given the central role of digital health literacy in promoting health care access, supporting self-management, and enabling social participation, dedicated research on individuals with serious mental illness is needed. Accordingly, we assessed key factors associated with digital health literacy among patients with serious mental illness. Specifically, we investigated whether mobile device proficiency, attitudes toward technology, and trust in online health information were associated with digital health literacy after adjustment for relevant sociodemographic characteristics.

## Methods

### Study Design

This descriptive cross-sectional study adhered to the STROBE (Strengthening the Reporting of Observational Studies in Epidemiology) guidelines (Checklist 1) to ensure transparent and comprehensive reporting.

### Settings and Participants

Participants were recruited, using convenience sampling, from 2 psychiatric outpatient clinics, 1 day-care center, and 4 halfway houses located in different administrative districts of Taipei City, Taiwan. These institutions provide community-based psychiatric rehabilitation services and were selected based on service diversity and institutional willingness to collaborate. Psychiatrists or nursing staff at each site assisted in identifying eligible participants.

Taipei City is a metropolitan area with extensive digital infrastructure. As of January 2025, more than 94% of households have internet access, and 87% of the population accesses the internet on personal mobile devices [24]. Recruitment was conducted across multiple service models and geographic districts to ensure sample heterogeneity within this urban context.

Eligible participants were aged 18 years or older; had received a diagnosis of schizophrenia or type I bipolar disorder by an independent psychiatrist in accordance with the *Diagnostic and Statistical Manual of Mental Disorders, Fifth Edition* criteria; were clinically stable; and were able to read, write, and provide written informed consent. Individuals with severe psychosis, cognitive impairment, intellectual disability, or an inability to communicate or write in Chinese were excluded.

A total of 284 potentially eligible individuals were approached. Among these individuals, 17 refused to participate. Of those who agreed to participate, 12 did not complete the questionnaire. The final sample comprised 255 individuals (attrition rate: 10.2%). A post hoc power analysis conducted with G*Power (version 3.1.9.7) indicated that a sample size of 255 individuals when 18 predictors were included yielded a statistical power of 98.3%, assuming a medium effect size (*f*²=15) and *α*=.05 [15,16].

### Data Collection

Data were collected between May 2024 and February 2025. The first author explained the study purpose and procedures to potential participants and provided an information sheet detailing the confidentiality measures. Individuals who provided written informed consent individually completed a paper-based anonymous questionnaire in a quiet room for approximately 30 minutes.

### Measures

Based on the multidimensional conceptual framework outlined in the *Introduction* section, 3 validated instruments were used: the eHEALS, chosen to assess digital health literacy; the 16-item Mobile Device Proficiency Questionnaire (MDPQ-16), chosen to measure operational mobile device skills; and the Attitudes Toward Computer/Internet Questionnaire (ATC/IQ), chosen to evaluate technology-related attitudes and self-efficacy.

### Demographic Data and Digital Device Use Questionnaire

Data on participants’ gender, age, education level, marital status, employment status, economic status, psychiatric diagnosis, illness duration, disability severity, and comorbidities were collected. Under Taiwan’s Disability Rights Protection Act, individuals with chronic psychiatric conditions may receive formal disability certification. For certification purposes, disability severity is determined based on occupational functioning, social functioning, and activities of daily living and is categorized as mild, moderate, severe, or profound. Certification requires evaluation at designated hospitals and periodic reassessment.

To assess digital behaviors, participants were asked about their preferred sources of health information, health-related topics of interest, mobile device ownership, internet access, concerns regarding internet-sourced health information, and the duration of internet use. Two single-item measures were developed to assess perceived barriers to digital engagement: (1) “How difficult is it for you to obtain health information from the internet?” and (2) “How difficult is it for you to trust health information on the internet?” Responses were rated on a 5-point Likert scale (1=not at all difficult; 5=extremely difficult), with higher scores indicating greater perceived difficulty. An expert panel consisting of 1 psychiatrist, 2 psychiatric nurses, and 2 individuals with schizophrenia evaluated the clarity, relevance, and contextual appropriateness of these items before questionnaire administration to ensure face validity.

### eHealth Literacy Scale

We assessed digital health literacy by using the 8-item eHEALS, which measures the perceptions of one’s ability to identify, evaluate, and apply health information obtained online [6]. Items are rated on a 5-point Likert-type scale (1=strongly disagree; 5=strongly agree), yielding total scores ranging from 8 to 40, with higher scores indicating greater literacy. The total scores were used in all analyses, and the mean total scores are reported for descriptive statistics. The scale demonstrated high internal consistency in this study (Cronbach *α*=0.95).

### 16-Item Mobile Device Proficiency Questionnaire

The proficiency of mobile device use was measured using the MDPQ-16, which contains 2 items from all 8 subscales of the 46-item MDPQ (ie, entertainment, communication, data and file storage, internet, mobile device basics, calendar, troubleshooting and software management, and privacy) [25]. Items were scored on a 5-point Likert-type scale (1=never tried; 5=very easily). Within each subscale, item scores were averaged; subscale means were then summed to generate a total score ranging from 8 to 40, with higher scores indicating greater proficiency. Cronbach *α* for the overall scale was 0.97, and subscale Cronbach *α* values ranged from 0.81 (entertainment) to 0.98 (calendar).

### Attitudes Toward Computer/Internet Questionnaire

We assessed attitudes toward digital technology by using the 10-item ATC/IQ modified by Choi and Dinitto [26]. This questionnaire comprises 2 subscales: self-efficacy (confidence in using digital devices or the internet) and interest (enjoyment and curiosity). Items are rated on a 5-point Likert-type scale (1=strongly disagree; 5=strongly agree). Item responses were averaged within each domain to calculate subscale scores. Higher scores indicate greater confidence or interest in using digital devices or the internet. Cronbach *α* values were 0.90, 0.81, and 0.89 for the self-efficacy subscale, interest subscale, and overall scale, respectively.

### Data Analysis

All data analyses were performed in SPSS (version 26.0; IBM). Statistical significance was defined as 2-tailed *P*<.05. Additionally, we calculated descriptive statistics to summarize demographic characteristics and digital behavior parameters. Continuous variables are presented as means and SDs, and categorical variables are presented as frequency (%) values.

Pearson correlation analyses were conducted to examine bivariate relationships among continuous variables. Assumptions of linearity and approximate normal distribution were assessed prior to performing the Pearson analysis. Correlation coefficients were interpreted according to Cohen guidelines (0.10=small, 0.30=moderate, and 0.50=large).

Univariate regression analyses were first conducted to explore the associations between digital health literacy (dependent variable) and potential predictors. Variables were included a priori in the multivariable generalized linear model based on theoretical considerations and empirical evidence. Predictors were grouped into four domains: (1) sociodemographic characteristics (age, educational level, income), (2) health-related characteristics (physical comorbidities), (3) digital access and usage behaviors (mobile device ownership, duration of mobile device use, regularity of internet use, and daily internet use duration), and (4) psychosocial factors (perceived difficulty in searching for digital health information, perceived difficulty in trusting digital health information, mobile device use proficiency, self-efficacy, and interest in digital health).

Multicollinearity was evaluated based on variance inflation factors, which ranged from 1.48 to 1.75, indicating no problematic collinearity. Regression estimates were reported as unstandardized coefficients with corresponding 95% CIs. All incomplete questionnaire responses were excluded before analysis, yielding a final dataset with no missing values.

### Ethical Considerations

The institutional review board of the principal investigator’s university approved this study (reference number A202405008). All participants provided written informed consent. Participation was voluntary, and individuals were informed that their decision to participate or withdraw would not affect their access to health care services. No researcher involved in data collection was responsible for the clinical care of participants to reduce the likelihood of social desirability bias. All data were deidentified, stored securely, and handled in accordance with institutional privacy and confidentiality policies. As compensation for their time, participants received a supermarket gift certificate valued at New Taiwan $200 (approximately US $6.50).

## Results

### Participant Characteristics

Table 1 summarizes participants’ demographic and clinical characteristics. The sample included 105 men and 150 women with a mean age of 50.4 (SD 12.6; range 20‐78) years. Most participants were single (n=222, 87.1%), had completed high school (n=116, 45.5%), were unemployed (n=187, 73.3%), and reported a general-level household income (n=151, 59.2%; approximately New Taiwan $1,137,000 [approximately US $35,800] per year in Taiwan). Among participants, 78.4% (n=200) had schizophrenia or a related disorder. The mean illness duration was 18.2 years (SD 11.7; range 1‐53), and 59.2% (n=151) of the participants had moderate illness severity. Additionally, 63.9% (n=163) of the participants reported having at least 1 physical comorbidity.

**Table 1.** Participant characteristics and digital device use patterns (n=255).

Variable	Values
Demographics	
Gender, n (%)	
Man	105 (41.2)
Woman	150 (58.8)
Age (y), mean (SD; range)	50.4 (12.6; 20-78)
Marital status, n (%)	
Single	222 (87.1)
Married	33 (12.9)
Education level, n (%)	
Elementary or junior high school	42 (16.5)
Senior high school	116 (45.5)
College or above	97 (38)
Employment status, n (%)	
Employed	68 (26.7)
Household income level, n (%)	
General	151 (59.2)
Low	104 (40.8)
Clinical factors	
Mental disorder, n (%)	
Schizophrenia and related disorders	200 (78.4)
Bipolar I disorder	55 (21.6)
Mental illness duration (y), mean (SD; range)	18.2 (11.8; 1-53)
Severity of disability, n (%)	
Mild	94 (36.9)
Moderate	151 (59.2)
Severe	10 (3.9)
Physical comorbidities^a^, n (%)	163 (63.9)
Digital use behaviors, n (%)	
Types of digital device used, yes^a^	213 (83.5)
Smartphone	183 (71.8)
Desktop computer	51 (20)
Laptop	25 (9.8)
Average duration of mobile device use (y), mean (SD; range)	14.9 (10.5; 1-41)
Average daily duration of internet use (h), mean (SD; range)	2.5 (2.7; 0-15)
Unlimited mobile data plan, n (%)	74 (29)
Regular internet user, yes, n (%)	185 (72.5)
Barriers, mean (SD; range)	
Difficulty in searching for digital health information^b^	2 (1.21; 1-5)
Difficulty in trusting digital health information^b^	2.59 (0.88; 1-5)

a Only the 3 most common items are listed.

b The data were collected only from regular internet users.

### Digital Device Use

Among the participants, 83.5% (n=213) reported owning at least 1 digital device. Smartphones were the most commonly owned devices (n=183, 71.8%). The average lifetime duration of digital device use was 14.9 (SD 10.5; range 1‐41) years, and the mean duration of daily device use was 2.5 (SD 2.7; range 0‐15) hours. Only 29% (n=74) of the participants had access to an unlimited mobile data plan. Approximately 72.5% (n=185) of the participants reported being regular internet users.

The participants reported low perceived difficulty in searching for digital health information (mean score 2.00, SD 1.21) and moderate difficulty in trusting such information (mean score 2.59, SD 0.88).

### Digital Health Literacy, Mobile Device Proficiency, and Attitudes

**Figure 1.**
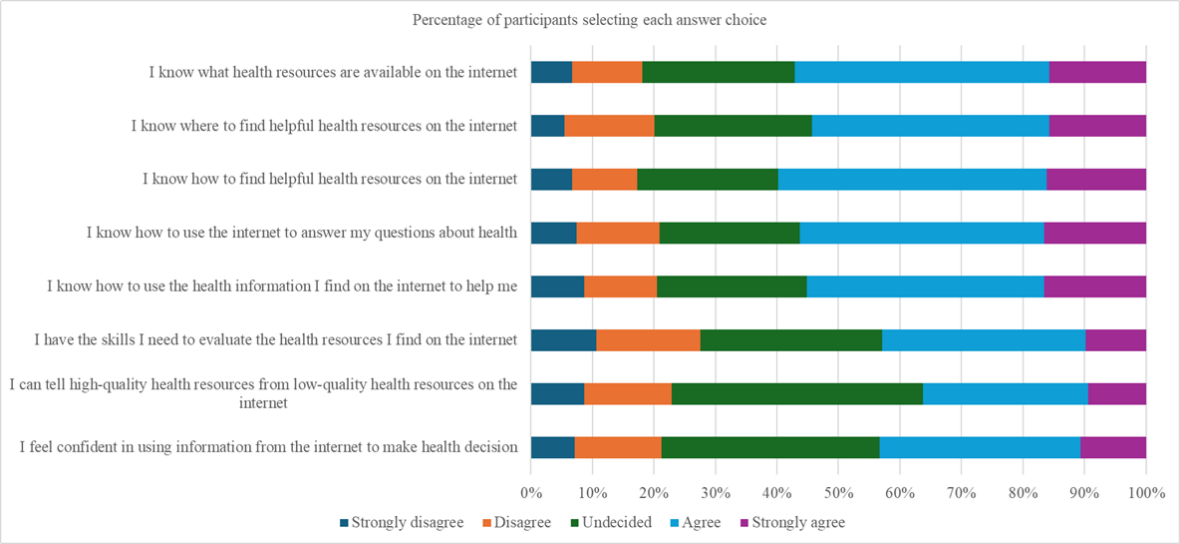
Response percentages for eHealth Literacy Scale (eHEALS) items across all participants (n=255).

Figure 1 illustrates the item-level distribution of responses to the eHEALS. Although many participants responded “agree” or “strongly agree” to all 8 items, a substantial proportion also chose the “undecided” option, indicating their uncertainty regarding several digital health competencies. As indicated in Table 2, the mean eHEALS score for overall perceived digital health literacy was 26.9 (SD 7.50; range 8‐40). Participants reported the highest confidence in locating helpful online health resources (“I know how to find helpful health resources on the internet”; mean score 3.5, SD 1.10). Confidence was the lowest for distinguishing the quality of online health information (“I can tell high-quality health resources from low-quality health resources of the internet”; mean score 3.1, SD 1.06). This item also received the highest proportion of undecided responses, suggesting limited perceived competence in critical appraisal skills.

**Table 2.** Descriptive statistics and correlations between study variables.

Variable	Scale	Score, mean (SD)	Digital health literacy, *r*	Mobile device proficiency, *r*	Self-efficacy, *r*
Digital health literacy	eHEALS^a^	26.9 (7.50)	—^b^	—	—
Mobile device proficiency	MDPQ^c^	23.3 (10.95)	0.52^d^	—	—
Self-efficacy	ATC/IQ^e^: efficacy	17.6 (4.68)	0.55^d^	0.63^d^	—
Interest	ATC/IQ: interest	18.7 (3.97)	0.49^d^	0.48^d^	0.70^d^

a eHEALS: eHealth Literacy Scale.

b Not applicable.

c MDPQ-16: 16-item Mobile Device Proficiency Questionnaire.

d *P*<.001.

e ATC/IQ: Attitudes Toward Computer/Internet Questionnaire.

The mean MDPQ score was 23.3 (SD 10.95; range 8‐40), suggesting considerable variation in mobile device proficiency within the sample. The “mobile device basics” domain received the highest score (mean 3.6, SD 1.49), and the “data and file storage” domain received the lowest score (mean 2.4, SD 1.56).

The mean overall ATC/IQ score was 3.6 (SD 0.80; range 1‐5), with the subscale mean scores for self-efficacy and interest of 3.5 (SD 0.94; range 1‐5) and 3.7 (SD 0.79; range 1‐5), respectively. These results indicate overall positive attitudes toward digital technology.

Correlation analyses revealed significant positive associations between digital health literacy and mobile device proficiency (*r*=0.52; *P*<.001), self-efficacy (*r*=0.55; *P*<.001), and interest (*r*=0.49; *P*<.001). Mobile device proficiency was positively correlated with interest (*r*=0.48; *P*<.001) and self-efficacy (*r*=0.63; *P*<.001). We also observed a positive association between the self-efficacy and interest ATC/IQ subscales (*r*=0.70; *P*<.001).

### Univariate Associations With Digital Health Literacy

Table 3 presents the univariate regression findings. Higher digital health literacy was significantly associated with younger age (*P*<.001), attainment of a college-level or higher degree (*P*=.004), general-level household income (*P*=.03), digital device ownership (*P*<.001), longer lifetime duration of device use (*P*<.001), longer daily internet use (*P*<.001), greater mobile device proficiency (*P*<.001), and more positive attitudes toward digital technologies (self-efficacy and interest; both *P*<.001).

Lower digital health literacy was significantly associated with physical comorbidities (*P*=.004), higher perceived difficulty in searching for health information online (*P*<.001), and lower trust in online health information (*P*<.001).

**Table 3.** Associations between key variables and digital health literacy.

Variable	*B* (SE; 95% CI)	*P* value
Age (y)	–0.145 (0.036; –0.216 to –0.074)	<.001^a^
Gender		
Woman vs man	–1.833 (0.946; –3.687 to 0.022)	.053
Marital status		
Married vs single	0.65 (1.396; –2.085 to 3.386)	.64
Education level		
College education or higher vs high school education or lower	2.748 (0.951; 0.884 to 4.611)	.004^b^
Employment status		
Yes vs no	2.058 (1.057; –0.015 to 4.13)	.05
Household income level		
General vs low	2.088 (0.946; 0.235 to 3.942)	.03^c^
Mental disorder		
Bipolar disorder vs schizophrenia	0.506 (1.139; –2.739 to 1.727)	.66
Mental illness duration (year)	–0.055 (0.04; –0.133 to 0.023)	.17
Disability		
Moderate or higher vs mild	–0.901 (0.971; –2.803 to 1.002)	.35
Physical comorbidities		
Yes vs no	–2.772 (0.963; –4.661 to –0.884)	.004^b^
Mobile device ownership^d^		
Yes vs no	4.255 (1.042; 2.212 to 6.297)	<.001^a^
Mobile device use duration (year)	0.184 (0.043; 0.099 to 0.268)	<.001^a^
Daily internet use duration (h)	0.624 (0.17; 0.291 to 0.957)	<.001^a^
Regular internet use		
Yes vs no	6.372 (0.977; 4.458 to 8.286)	<.001^a^
Difficulty in searching for digital health information	–3.103 (0.348; –3.784 to –2.422)	<.001^a^
Difficulty in trusting digital health information	–2.809 (0.532; –3.852 to –1.766)	<.001^a^
Mobile device use proficiency	0.355 (0.037; 0.283 to 0.428)	<.001^a^
Self-efficacy	4.43 (0.419; 3.609 to 5.251)	<.001^a^
Interest	4.626 (0.516; 3.614 to 5.637)	<.001^a^

a *P*<.001.

b *P*<.01.

c *P*<.05.

d Ownership of a smartphone or laptop.

### Multivariable Regression Analysis

Table 4 presents the multivariable regression results. After adjustment for covariates, 4 variables remained significantly associated with digital health literacy. Digital health literacy was negatively associated with both the perceived difficulty of searching for online health information (*B*=−1.533, 95% CI −2.350 to −0.717; *P*<.001) and distrust in online health information (*B*=−0.986, 95% CI −1.916 to −0.056; *P*=.04). Mobile device proficiency (*B*=0.144, 95% CI 0.008‐0.281; *P*=.04) and self-efficacy (*B*=1.777, 95% CI 0.376‐3.177, *P*=.01) were positively associated with digital health literacy. Interest in digital technology demonstrated a marginal association with digital health literacy (*P*=.050).

**Table 4.** Multivariable linear regression results for factors associated with digital health literacy.

Variable	*B* (SE; 95% CI)	*P* value
Age	0.07 (0.382; –0.005 to 0.145)	.07
Education level		
College education or higher vs high school education or lower	–1.331 (0.83; –2.958 to 0.296)	.11
Household income level		
General vs low	–0.384 (0.827; –2.005 to 1.236)	.64
Physical comorbidities		
Yes vs no	–1.199 (0.806; –2.778 to 0.381)	.14
Mobile device ownership^a^		
Yes vs no	–2.386 (1.659; –5.638 to 0.098)	.14
Mobile device use duration (y)	0.052 (0.48; –0.042 to 0.146)	.27
Regular internet use		
Yes vs no	3.998 (5.458; –6.698 to 14.694)	.54
Daily internet use duration (h)	–0.236 (0.161; –0.553 to 0.08)	.14
Difficulty in searching for digital health information	–1.533 (0.417; –2.35 to –0.717)	<.001^b^
Difficulty in trusting digital health information	–0.986 (0.474; –1.916 to –0.056)	.04^c^
Mobile device use proficiency	0.144 (0.695; 0.008 to 0.281)	.04^c^
Self-efficacy	1.777 (0.715; 0.376 to 3.177)	.01^c^
Interest	1.45 (0.739; 0.002 to 2.898)	.05

a Ownership of a smartphone or laptop.

b *P*<.001.

c *P*<.05.

## Discussion

### Principal Findings

We examined digital health literacy among patients with serious mental illness and identified factors associated with variation in literacy levels. Although most of the participants owned digital devices and were regular internet users, their overall digital health literacy and mobile device proficiency were moderate. Mobile device proficiency, self-efficacy, and interest in technology were positively associated with digital health literacy, whereas the perceived difficulty of searching for and distrust in online health information were negatively associated with digital health literacy. These findings highlight modifiable targets for improving digital inclusion, underscoring the need for tailored strategies to support meaningful engagement with online health resources.

### Comparison With Prior Work

The mean eHEALS score was 26.9, indicating that participants perceived themselves as having moderate digital health literacy. This result aligns with scores reported for individuals with serious mental illness in the United Kingdom [16] and for individuals with schizophrenia in Finland [15]. Conversely, higher digital literacy scores were reported for individuals with bipolar disorder in the United States [17]. This discrepancy may be attributable to variations in clinical characteristics, symptom profiles, and cognitive functioning. Individuals with psychosis often experience concentration difficulties, disorganized thinking, and paranoid ideation, which can impede their ability to effectively search for, interpret, and apply online health information [10,23]. Furthermore, disparities in digital engagement are correlated with demographic factors, including age, education, and employment status [8,18,19].

The substantial variability in eHEALS scores in our sample (range 8‐40) suggests that digital health literacy is heterogeneous among patients with serious mental illness, shaped by intersecting sociodemographic, clinical, and experiential factors. Such variability reinforces the value of tailored interventions that address different levels of need—from foundational digital skills to advanced competencies, such as the appraisal of online information.

Mobile device proficiency significantly predicted digital health literacy, consistent with findings that core digital skills (eg, connecting to Wi-Fi, navigating search engines, managing privacy settings, and troubleshooting) are essential for effective online engagement [25]. Although their rate of device ownership was high, our participants demonstrated poorer mobile device proficiency than that reported in another study involving individuals with schizophrenia [27]. Similarly, another study demonstrated that device ownership does not guarantee digital competence among individuals with serious mental illness [10]. A multicountry qualitative study further indicated that limited digital competence deterred patients with serious mental illness from engaging in mental health programs through digital platforms [28].

Widespread and accessible digital infrastructure (as in Taipei City) does not automatically lead to meaningful digital engagement for individuals with serious mental illness. In our sample, smartphone ownership was common; however, few (n=74, 29%) participants reported having access to an unlimited mobile data plan. Many of the participants relied on family members to obtain smartphones, which they used primarily to maintain contact and ensure their safety rather than for general digital participation. These devices were frequently older models passed down from relatives or purchased due to the declining availability of basic keypad phones. Financial constraints also limited participants’ ability to afford unlimited data plans, restricting their continuous internet access. Consequently, device use largely centered on vocal communication rather than on active information seeking or interactive digital engagement. Studies of older adults [29] and young adults with chronic illness [30] have similarly identified a gap between digital access and meaningful use. Structural and relational factors may collectively contribute to heterogeneity in eHEALS-measured digital health literacy by limiting experiential exposure to digital environments and reducing opportunities to build confidence in locating, evaluating, and applying online health information.

According to Bandura’s social cognitive theory, self-efficacy is defined as an individual’s self-perceived ability to effectively perform tasks [31]. In our study, technological self-efficacy was positively associated with digital health literacy. Individuals who believe in their ability to navigate digital environments typically also engage in and sustain health information–seeking behaviors, even when facing challenges [32]. Interest in technology (a related motivational factor) was also positively correlated with digital health literacy. User attitudes toward digital technology are shaped by their perceptions of that technology’s usefulness and ease of use [26]. Perceived usefulness and ease of use in turn influence individuals’ motivation to explore, adopt, and maintain digital behaviors [33]. Interest also deepens cognitive engagement, supports sustained attention, enhances information processing, and facilitates learning [34]. These motivational factors are critical for meaningful engagement, especially when attitudes toward technology are neutral or ambivalent [4]. Thus, strategies to foster curiosity and personal relevance may be as critical as technical skills training.

The perceived difficulty of searching for and evaluating digital health information was negatively associated with digital health literacy. Although participants frequently used mobile devices, their use centered on communication and entertainment rather than on health information seeking, a pattern also observed in other studies [22]. Time spent online does not equate to competence. Effective digital engagement requires search strategy knowledge and skills, familiarity with credible sources, and general health literacy [35,36]. Therefore, digital access alone is insufficient to ensure meaningful engagement with health information; rather, cognitive, informational, and motivational support are necessary to promote the skills, confidence, and critical judgment required to overcome digital exclusion and effectively use digital health resources.

Participants’ limited trust in digital health information formed a major literacy barrier, indicating that trust is a central component of digital health literacy. The eHealth Literacy 3.0 framework emphasizes that literacy extends beyond information retrieval, encompassing the ability to safely and meaningfully engage with digital technologies to achieve health goals [11]. Our participants reported concerns regarding inconsistencies between sources, commercially driven content, and discrepancies between online information and their lived experiences, which align with other research indicating that the quality of publicly available mental health resources is highly heterogeneous [17]. The ongoing “infodemic” and the variable quality and transparency of publicly available mental health applications have further amplified these concerns [37].

Individuals with serious mental illness often rely heavily on health care professionals as trusted sources of information [15,22]. This reliance reinforces therapeutic relationships but may inadvertently reduce independent engagement with digital resources. To bridge this gap, strategies to strengthen and scaffold trust should extend beyond simple referrals. Structured guided programs, such as Digital Opportunities for Outcomes in Recovery Services, have demonstrated promise in connecting patients with “digital navigators”—trained staff or peers with lived experience who provide hands-on assistance in distinguishing between safe and high-risk digital tools [38]. Structured engagement strategies may help patients translate their perceived self-efficacy into functional digital competence [39]. Additionally, clinician-curated lists of vetted digital resources may serve as structured entry points into the digital environment, reducing patient uncertainty and fostering confidence in information credibility [17]. Such interventions do not merely provide access to trustworthy resources but instead empower individuals to develop and sustain the critical appraisal skills necessary for long-term self-management.

### Implications for Practice

Health care professionals play pivotal roles in supporting digital inclusion and self-management among individuals with serious mental illness. Given the considerable variability in digital health literacy we observed within this population, health care professionals should incorporate routine assessment of digital health literacy using brief validated tools (eg, the eHEALS) into current clinical workflows to identify individuals at risk of digital exclusion. Such assessments can allow health care professionals to stratify support and allocate resources according to each individual’s skill-related and motivational needs.

Targeted interventions should address both technical and clinical barriers. For individuals with low digital device proficiency, structured digital skills training may increase smartphone competency and autonomy. Confidence-building and guided practice sessions may support individuals with low self-efficacy, and clinician-curated navigation of trustworthy online resources may mitigate distrust in digital health information. Interventions must also account for symptom-related barriers associated with serious mental illness and can be embedded within established psychoeducation groups, case management services, or community-based rehabilitation programs to support feasibility and sustainability.

Policymakers and digital health program designers should recognize digital health literacy as a crucial determinant of health equity and prioritize user-centered strategies that foster empowerment. In addition to technical skills training, coaching or peer-led support for self-efficacy may bridge the gap between perceived and actual competence. Accessible, culturally appropriate, and plain-language digital platforms must be available to ensure sustained engagement. Finally, nondigital engagement options should be maintained to prevent unintended exclusion and ensure that mental health care systems remain inclusive and responsive to diverse patient needs.

### Strengths and Limitations

This study makes a timely and valuable contribution to the growing field of digital health by focusing on individuals with serious mental illness, who remain largely underrepresented in such research. We recruited a relatively large and diverse sample across multiple mental health settings in Taiwan to provide a nuanced and context-specific understanding of digital health literacy within this vulnerable group. Our use of well-validated instruments, including the eHEALS, MDPQ-16, and ATC/IQ, strengthened the methodological rigor and credibility of the findings. Furthermore, we constructed multivariate generalized linear models adjusted for key sociodemographic variables to identify modifiable predictors (ie, mobile use proficiency, self-efficacy, and interest in technology) and key barriers (ie, difficulty in searching for information and distrust in online content) related to digital health literacy. We are among the first scholars to examine the hypothesis that both technical skills and psychosocial determinants influence the digital health literacy of individuals with serious mental illness. Our findings provide a foundation for the development of tailored, equity-focused interventions to expand access to digital health tools and foster meaningful, confident engagement with online health information.

Several limitations of this study warrant consideration. First, the cross-sectional design inhibits our ability to make causal inferences regarding the relationship between the identified predictors and digital health literacy. Second, we performed convenience sampling within metropolitan Taipei, limiting the generalizability of our findings to rural, inpatient, or acutely symptomatic populations. Interregional differences in digital infrastructure and service availability may affect the generalizability of the findings. Third, we measured digital health literacy by using a self-reported instrument that captured perceived rather than objective competence. The discrepancy observed between device ownership and operational proficiency suggests that performance-based assessments would provide valuable complementary data. Finally, we did not directly assess clinical variables that may have contributed to residual confounding, such as symptom severity, cognitive functioning, and medication effects.

### Recommendations for Future Research

Future longitudinal or experimental studies are warranted to clarify the directionality and causality of relationships between digital health literacy and its modifiable predictors, such as mobile device proficiency and self-efficacy. Additionally, performance-based assessments and behavioral measures of online engagement could more objectively capture digital competencies, overcoming the limitations of self-reported data. Furthermore, scholars should investigate factors influencing digital health literacy that were not included in this study, such as the severity of psychiatric symptoms, participation in digital training programs, and engagement with digital health services provided by mental health professionals.

### Conclusions

Digital health literacy among individuals with serious mental illness in Taiwan remains suboptimal, despite relatively high rates of digital device ownership and internet access. The wide variation in eHEALS scores indicates that heterogeneity in digital health literacy within this population reflects a broad spectrum of digital engagement. Multivariate linear regression analysis identified mobile device use proficiency, self-efficacy, and interest in technology as modifiable predictors of literacy, whereas the difficulty of searching for online health information and distrust in such information were notable barriers. Tailored, multilevel programs that address both foundational digital skills and higher-order competencies, such as critical appraisal and digital trust, are required. Future research should incorporate more diverse and representative samples and objective assessments to facilitate the development of scalable, inclusive digital literacy interventions.

## Supplementary material

10.2196/88700 Checklist 1 STROBE checklist.

## Abbreviations

ACT/IQ: Attitudes Toward Computer/Internet Questionnaire
eHEALS: eHealth Literacy Scale
MDPQ-16: 16-item Mobile Device Proficiency Questionnaire
STROBE: Strengthening the Reporting of Observational Studies in Epidemiology
